# Virginia Medical Society

**Published:** 1890-11

**Authors:** 


### VIRGINIA MEDICAL SOCIETY.

### Rockbridge, Alum Springs.

Meeting, September 4th.

Dr. Edwin Ricketts, of Cincinnati, read a paper on Early Exploratory Incision as an Aid to the Diagnosis of some Surgical Diseases of the Abdominal Cavity.

He had found it difficult in many cases to make a diagnosis previous to exploratory incision.

To open the abdomen was easy enough, but afterwards to do *always* the best thing and that *promptly*, knowing when to end at exploration, bearing in mind that half-completed surgical procedures are *rarely ever excusable*, these are of greatest consideration.

He did not deny that a diagnosis cannot be made previous to an incision in some cases, but in a majority we cannot make out with any degree of certainty until, "Thomas-like," we first *see* and *feel* the condition present.

He claimed that the one from the outside that is always sure of what is on the inside is the one with his *occasional* operations is placed in a humiliating attitude before his guests, and is liable to attempt the completion of an operation that may be *wholly unwarrantable*. The undue conservative physician, with his free use of opiates, has grave responsibilities, for the reason that his prompt efforts for the relief of pain too often postpones the recognition of needed surgical interference.

He reports briefly eleven cases, coming under his observation, where exploratory incision was necessary to diagnosis.

